# Surgical Clipping versus Endovascular Intervention for the Treatment of Subarachnoid Hemorrhage Patients in New York State

**DOI:** 10.1371/journal.pone.0137946

**Published:** 2015-09-11

**Authors:** Kimon Bekelis, Symeon Missios, Shannon Coy, Redi Rahmani, Robert J. Singer, Todd A. MacKenzie

**Affiliations:** 1 Section of Neurosurgery, Dartmouth-Hitchcock Medical Center, Lebanon, NH, United States of America; 2 Department of Neurosurgery, Louisiana State University Health Sciences Center, Shreveport, LA, United States of America; 3 Geisel School of Medicine at Dartmouth, Hanover, NH, United States of America; 4 Department of Medicine, Dartmouth-Hitchcock Medical Center, Lebanon, NH, United States of America; 5 Department of Community and Family Medicine, Dartmouth-Hitchcock Medical Center, Lebanon, NH, United States of America; 6 The Dartmouth Institute for Health Policy and Clinical Practice, Lebanon, NH, United States of America; St Michael's Hospital, University of Toronto, CANADA

## Abstract

**Object:**

Randomized trials have demonstrated a survival benefit for endovascular treatment of ruptured cerebral aneurysms. We investigated the association of surgical clipping and endovascular coiling with outcomes in subarachnoid hemorrhage (SAH) patients in a real-world regional cohort.

**Methods:**

We performed a cohort study involving patients with ruptured cerebral aneurysms, who underwent surgical clipping, or endovascular coiling from 2009–2013 and were registered in the Statewide Planning and Research Cooperative System (SPARCS) database. An instrumental variable analysis was used to investigate the association of treatment technique with outcomes.

**Results:**

Of the 4,098 patients undergoing treatment, 2,585 (63.1%) underwent coiling, and 1,513 (36.9%) underwent clipping. Using an instrumental variable analysis, we did not identify a difference in inpatient mortality [marginal effect (ME), -0.56; 95% CI, -1.03 to 0.02], length of stay (LOS) (ME, 1.72; 95% CI, -3.39 to 6.84), or the rate of 30-day readmissions (ME, -0.30; 95% CI, -0.82 to 0.22) between the two treatment techniques for patients with SAH. Clipping was associated with a higher rate of discharge to rehabilitation (ME, 0.63; 95% CI, 0.24 to 1.01). In sensitivity analysis, mixed effect regression, and propensity score adjusted regression models demonstrated identical results.

**Conclusions:**

Using a comprehensive all-payer cohort of patients in New York State presenting with aneurysmal SAH we did not identify an association of treatment method with mortality, LOS or 30-day readmission. Clipping was associated with a higher rate of discharge to rehabilitation.

## Introduction

Cerebral aneurysm rupture and subarachnoid hemorrhage (SAH) have catastrophic consequences, with major morbidity and mortality.[1–3] Surgical clipping or endovascular coiling can be used to secure the aneurysm and prevent further bleeding.[1] In 2002, the International Study for Aneurysm Treatment (ISAT)[4] demonstrated that the minimally invasive option of coiling is associated with improved mortality one year postoperatively. These results, although initially highly controversial,[5] were subsequently confirmed in a single-center randomized trial in the United States.[6] Following the publication of these results, endovascular treatment of ruptured cerebral aneurysms has become the predominant option in SAH.[7] However, the relative effectiveness of clipping and coiling has not been studied extensively in the community. This is particularly important, given the concerns that have been raised about the lack of unified certification criteria for endovascular practitioners.[2, 3]

However, observational studies attempting to answer this question are subject to selection bias. Patients included in retrospective analyses were selected for either procedure in advance. This selection typically reflects the different preferences and backgrounds of the treating physicians, as well as specific patient characteristics, and anatomic information such as aneurysm size, shape and location. Administrative databases lack such granularity, thus limiting the ability to control for such confounders. This introduces significant unmeasured confounding. There has been no prior study attempting to account for these limitations through different analytic approaches in an adult cohort of all ages.

We used the New York Statewide Planning and Research Cooperative System (SPARCS)[8] to study the association of treatment technique with mortality, discharge to rehabilitation, 30-day readmission, and length of stay for patients undergoing surgical clipping or endovascular coiling for ruptured cerebral aneurysms. An instrumental variable analysis was used to control for unmeasured confounding and simulate the effect of randomization.

## Methods

### New York Statewide Planning and Research Cooperative System (SPARCS)

The Dartmouth Committee for Protection of Human Subjects has approved this analysis as this is de-identified database. All patients with ruptured cerebral aneurysms who were registered in the SPARCS (New York State Department of Health, Albany, NY)[8] database between 2009 and 2013 were included in the analysis. For these years, SPARCS contains patient-level details for every hospital discharge, ambulatory surgery, and emergency department admission in New York State as coded from admission and billing records. This is an all payer database. SPARCS is different from other administrative databases by providing information on the specific physician, and hospital where the patient received treatment. Additionally, the ZIP code of the patient’s residence is available to the researchers. In our analysis, we used the limited version (not the public use file) of the dataset. More information about SPARCS is available at https://www.health.ny.gov/statistics/sparcs/.

### Cohort Definition

In order to establish the cohort of patients, we used *International Classification of Disease-9-Clinical Modification* (ICD-9-CM) codes to identify patients in the registry who underwent surgical clipping (ICD-9-CM procedure code 39.51) or endovascular coiling [patient with one of the following ICD-9-CM procedure codes: 39.52 (should also have a code 88.41 and no 39.51 during the same hospitalization), 39.72, 39.75, 39.76; all these codes correspond to endovascular treatments of cerebral aneurysms and have been used before in the literature][2] for ruptured (ICD-9-CM diagnosis code 430) cerebral aneurysms between 2009 and 2013. To avoid bias arising from including patients in the cohort at multiple time points, we only considered the first intervention each patient received and measured the outcomes associated with that hospitalization. If patients underwent a different intervention at a later date for a separate aneurysm we did not reconsider them for the analysis, since they were already censored.

### Outcome variables

The primary outcome variable was mortality during the initial hospitalization after treatment of a ruptured cerebral aneurysm. Secondary outcomes were length-of-stay (LOS) during the initial hospitalization, the rate of discharge to rehabilitation facility (any facility other than the patient’s home), and 30-day post-discharge readmission to any hospital.

### Exposure variables

The primary exposure variable was the treatment method (surgical clipping versus endovascular coiling).

Covariates (S1 Table) used for risk-adjustment were age, gender, race (African-American, Hispanic, Asian, Caucasian, other), and insurance (private, Medicare, Medicaid, uninsured, other). The comorbidities used for risk adjustment were diabetes mellitus (DM), smoking, chronic lung disease, hypertension, hypercholesterolemia, peripheral vascular disease (PVD), congestive heart failure (CHF), coronary artery disease (CAD), history of ischemic stroke, transient ischemic attack (TIA), alcohol abuse, obesity, chronic renal failure (CRF), and coagulopathy. Only variables that were defined as “present on admission” were considered part of the patient’s preadmission comorbidity profile.

### Statistical analysis

The association of treatment technique with our outcome measures was examined in a multivariable setting. Patients undergoing surgical clipping, or endovascular coiling in our cohort were selected for either procedure based on provider and patient preferences in a non-random way, before our study was executed. In order to account for this unmeasured confounding, and to simulate the effect of randomization, we used an instrumental variable analysis, an econometric technique.[9] This analysis controls for unmeasured confounders (such as SAH grade, or aneurysm location) by creating randomization on the treatment method. It is expected that this approach will balance the comparison groups in terms of variables we cannot measure. Such comparisons with instrumental variable analysis have been published before for different anesthesia techniques,[10] partial versus radical nephrectomy,[11] and stroke center versus no stroke center care.[12] In all these cases, but particularly in the last example (which used the same database as our study) the authors did not have data on the baseline NIH stroke scale or neurologic status of the patients. This was the reason they used an instrumental variable analysis.

The regional ratio of coiling (county level coiling ratio—defined as the number of coiled patients divided by the total number of interventions for cerebral aneurysms in a county) was used as an instrument. The regional ratio of a procedure has been used before to create pseudorandomization on the treatment method, using an instrumental variable analysis.[13] A good instrument is not associated with the outcome other than through the exposure variable of interest (a requirement known as the exclusion restriction criterion).[13] In our case it is unlikely that the regional ratios of coiling would be associated with treatment mortality in any way other than the choice of treatment. This has been accepted for similar instruments (regional ratio of a treatment) in prior literature.[10–12] A two stage least squares (2SLS) method was used for the calculation of the coefficients. The value of the F statistic in the first stage of the 2SLS approach was 30, which is consistent with a strong instrument (F statistic>10), based on a practical rule, published before in the literature.[9]

A probit regression was used for the categorical outcomes (mortality, discharge to rehabilitation, 30-day readmission),[14] and a linear regression for the linear outcomes (LOS). The covariates used for risk adjustment in these models were: age, gender, race, insurance, hospital ID, and all the comorbidities mentioned previously. Since the coefficients produced by the probit function are not interpretable, we used the marginal effects of our independent variables instead. The marginal effects are the partial derivatives of the coefficients, and reflect the change in the probability of the dependent variable, for 1 unit change in the independent variable, at the average value of all other covariates.

In order to demonstrate the robustness of our data in a sensitivity analysis, we used standard techniques to account for measured confounding while accounting for clustering at the hospital level. For categorical outcomes we used a logistic regression model with hospital name as a random effects variable, while controlling for all the covariates mentioned previously. In an alternative way to control for confounding, we used a propensity adjusted (with deciles of propensity score) logistic regression model. We calculated the propensity score of coiling, using a separate logistic regression model, adjusting for all the covariates mentioned previously. For continuous outcomes, similar analyses were conducted using linear models. Logarithmic transformation of the values of LOS yielded identical results and is therefore not reported further.

Regression diagnostics were used for all models. The number needed to treat (NNT) was calculated when appropriate. All results are based on two sided tests, and the level of statistical significance was set at 0.05. This study, based on 4,098 patients, has sufficient power (90%) at a 5% type I error rate to detect differences in mortality, as small as 10.4%. Statistical analyses were performed using Stata version 13 (StataCorp, College Station, TX), the 64-bit version of R.3.1.0 (R Foundation for Statistical Computing), and SPSS version 22 (IBM, Armonk, NY).

## Results

### Patient characteristics

In the selected study period there were 4,098 patients undergoing treatment for ruptured cerebral aneurysms (mean age was 53.8 years, with 59.6% females) who were registered in SPARCS, 2,585 (63.1%) underwent endovascular coiling, and 1,513 (36.9%) underwent surgical clipping. The characteristic of the two cohorts at baseline can be seen in Table 1.

**Table 1:** Patient characteristics.

		All Patients		Coiled		Clipped		
		N = 4098		N = 2585		N = 1513		
		Mean	SD	Mean	SD	Mean	SD	P-Value
Age		53.81	14.25	54.25	14.73	53.05	13.37	<0.0001
		**N**	**%**	**N**	**%**	**N**	**%**	**P-Value**
Gender	F	2765	59.6%	1714	66.3%	1051	69.5%	0.038
	M	1333	28.7%	871	33.7%	462	30.5%	
Diabetes Mellitus	-	3686	79.4%	2318	89.7%	1368	90.4%	0.452
	+	412	8.9%	267	10.3%	145	9.6%	
Smoking	-	3039	65.5%	1903	73.6%	1136	75.1%	0.318
	+	1059	22.8%	682	26.4%	377	24.9%	
Obesity	-	3846	82.8%	2418	93.5%	1428	94.4%	0.312
	+	252	5.4%	167	6.5%	85	5.6%	
Transient Ischemic Attack	-	3931	84.7%	2493	96.4%	1438	95.0%	0.033
	+	167	3.6%	92	3.6%	75	5.0%	
Ischemic Stroke	-	4016	86.5%	2532	97.9%	1484	98.1%	0.818
	+	82	1.8%	53	2.1%	29	1.9%	
Coronary Artery Disease	-	3709	79.9%	2328	90.1%	1381	91.3%	0.205
	+	389	8.4%	257	9.9%	132	8.7%	
Chronic Obstructive Pulmonary Disease	-	3642	78.4%	2307	89.2%	1335	88.2%	0.328
	+	456	9.8%	278	10.8%	178	11.8%	
Congestive Heart Failure	-	3862	83.2%	2421	93.7%	1441	95.2%	0.037
	+	236	5.1%	164	6.3%	72	4.8%	
Coagulopathy	-	3998	86.1%	2518	97.4%	1480	97.8%	0.463
	+	100	2.2%	67	2.6%	33	2.2%	
Chronic Renal Failure	-	4083	87.9%	2576	99.7%	1507	99.6%	0.794
	+	15	0.3%	9	0.3%	6	0.4%	
Hypertension	-	1827	39.3%	1194	46.2%	633	41.8%	0.007
	+	2271	48.9%	1391	53.8%	880	58.2%	
Hypercholesterolemia	-	3362	72.4%	2108	81.5%	1254	82.9%	0.292
	+	736	15.9%	477	18.5%	259	17.1%	
Alcohol	-	3890	83.8%	2468	95.5%	1422	94.0%	0.039
	+	208	4.5%	117	4.5%	91	6.0%	
Peripheral Vascular Disease	-	3962	85.3%	2484	96.1%	1478	97.7%	0.007
	+	136	2.9%	101	3.9%	35	2.3%	

### Inpatient mortality

Overall, 117 (7.7%) inpatient deaths were recorded after clipping and 264 (10.2%) after coiling (Table 2). Clipping was associated with decreased mortality in comparison to coiling (OR, 0.73; 95% CI, 0.58 to 0.91) in unadjusted analysis. Likewise, there was no association of treatment technique with mortality (ME, -0.56; 95% CI, -1.03 to 0.02) after using a probit regression with instrumental variable analysis (Table 3). This persisted in a mixed effects logistic regression model (OR, 0.88; 95% CI, 0.69 to 1.14) and a propensity score adjusted model (OR, 0.83; 95% CI, 0.65 to 1.04).

**Table 2:** Outcomes.

	Total	Coiled	Clipped	P-value
Death, %	381 (9.29%)	264 (10.21%)	117 (7.73%)	0.008
Discharge to rehabilitation, %	2272 (55.44%)	1324 (51.22%)	948 (62.66%)	<0.0001
30-day readmission, %	287 (7.00%)	179 (6.92%)	108 (7.14%)	0.796
Length of stay, SD	17 (14)	16 (13)	18 (13)	<0.0001

SD: standard deviation.

**Table 3:** Multivariable models examining the association of surgical clipping with outcomes.

	Inpatient Mortality		Discharge to rehabilitation		30-day readmission		Length-of-stay§	
	**ME (95% CI)**	**P-value**	**ME (95% CI)**	**P-value**	**ME (95% CI)**	**P-value**	**ME (95% CI)**	**P-value**
Instrumental variable analysis*	-0.56 (-1.03 to 0.02)	0.130	0.63 (0.24 to 1.01)	<0.001	-0.30 (-0.82 to 0.22)	0.259	1.72 (-3.39 to 6.84)	0.509
	**OR (95% CI)**	**P-value**	**OR (95% CI)**	**P-value**	**OR (95% CI)**	**P-value**	**Beta (95% CI)**	**P-value**
Mixed effects logistic regression⌘	0.88 (0.69 to 1.14)	0.200	1.65 (1.39 to 1.95)	<0.001	1.05 (0.81 to 1.38)	0.694	1.26 (0.10 to 2.42)	0.334
Propensity score adjusted logistic regression¶	0.83 (0.65 to 1.04)	0.110	1.69 (1.45 to 1.96)	<0.001	1.05 (0.82 to 1.35)	0.707	1.16 (-0.02 to 2.33)	0.070

ME: marginal effects; CI: confidence intervals; OR: odds ratio.

*County coiling ratio was used as an instrument of coiling.

^⌘^Hospital ID was used as a random effects variable.

^¶^The propensity score was calculated using the following variables: sex, race, insurance, medical comorbidities.

^§^All regressions were based on linear models.

### Discharge to rehabilitation

Overall, 948 (62.7%) were discharged to rehabilitation after clipping and 1,324 (51.2%) after coiling (Table 2). Clipping was associated with an increased rate of discharge to rehabilitation in comparison to coiling (OR, 1.54; 95% CI, 1.34 to 1.78) in the unadjusted analysis. This persisted (ME, 0.63; 95% CI, 0.24 to 1.01) after using a probit regression with instrumental variable analysis (Table 3). We found similar results in a mixed effects logistic regression model (OR, 1.65; 95% CI, 1.39 to 1.95) and a propensity score adjusted model (OR, 1.69; 95% CI, 1.45 to 1.96). This corresponded to 9 patients needed to be treated with coiling to prevent one discharge to rehabilitation.

### 30-day readmission

Overall, 108 (7.1%) were readmitted within 30-days after clipping and 179 (6.9%) after coiling (Table 2). Clipping was not associated with increased rate of 30-day readmission in comparison to coiling (OR, 1.03; 95% CI, 0.80 to 1.32) in the unadjusted analysis. Similarly, there was no association (ME, -0.30; 95% CI, -0.82 to 0.22) after using a probit regression with instrumental variable analysis (Table 3). We found similar results in a mixed effects logistic regression model (OR, 1.05; 95% CI, 0.81 to 1.38) and a propensity score adjusted model (OR, 1.05; 95% CI, 0.82 to 1.35).

### Length-of-stay

The average LOS was 18 days (SD 13) after clipping, and 16 days (SD 13) after coiling (Table 2). Clipping was not associated with increased LOS in comparison to coiling (beta, 1.00; 95% CI, -1.14 to 1.16) in the unadjusted analysis. This persisted (ME, 1.72; 95% CI, -3.39 to 6.84) after using a linear regression with instrumental variable analysis (Table 3). We found similar results in a mixed effects linear regression model (beta, 1.26; 95% CI, -0.10 to 2.42) and a propensity score adjusted linear regression model (beta, 1.16; 95% CI, -0.02 to 2.33).

## Discussion

Using a comprehensive all-payer cohort of aneurysmal SAH patients in New York State we did not identify an association of treatment method with mortality, LOS, or 30-day readmission. Clipping was associated with higher rate of discharge to rehabilitation. Our results were robust when considering several advanced observational techniques to account for measured and unmeasured confounders. Endovascular coiling has seen explosive growth in recent years, especially after the publication of randomized trials supporting that it offers a survival benefit, in comparison to clipping, for patients with SAH.[7] However, the relative effectiveness of these two treatment interventions for ruptured cerebral aneurysms in the community, has not been established yet.[2, 3]

Several randomized trials have demonstrated short and long-term benefits of coiling in comparison to clipping for patients with ruptured cerebral aneurysms. Molyneux et al,[4] in their landmark ISAT study, demonstrated that 30.6% of patients undergoing clipping were dead or dependent one year after intervention, in comparison to 23.7% after coiling. Long-term follow up results of this trial demonstrated persistence of this survival benefit 5 years postoperatively, despite the increased risk of rebleeding for coiled aneurysms.[15] However, survivors undergoing either treatment had similar neurologic outcomes. Initial criticism for the ISAT trial focused on the selection of anterior circulation aneurysms and the inclusions of mainly European centers.[5] A single center trial in the United States, designed to address these potential short-comings, confirmed the superiority of coiling in 1-year postoperative survival.[6] The non-invasive nature of endovascular techniques, and these encouraging results have led to their explosive growth. However, results of well-designed, controlled trials do not always translate to real-world effectiveness.[16, 17] Rigorous studies of the latter for SAH patients are lacking.

Traditional techniques used in most observational studies to control for measured confounding are not ideal for such a study. The selection of patients for either treatment prior to the analysis introduces significant unmeasured confounding. Patients may be selected for coiling because of favorable anatomy, aneurysm location, or general health. Physician or patient preference, as well as provider training and specialty might affect that decision too. Not accounting for this dimension of confounding puts the robustness of the findings of an observational study into question. Our study, purposefully addressing this potential bias, utilized an econometric technique, to account for unmeasured confounding and simulate pseudo-randomization. We used regional coiling ratio, a well-established type of instrument,[13] for our analytic strategy.

The present analysis did not demonstrate a difference in inpatient mortality between the patients undergoing surgical clipping and endovascular coiling. This is not in accordance with the survival benefit of clipping demonstrated by all prior randomized trials.[4, 6, 15] It is likely that the widespread availability of coiling, and the use of this technique by potentially less experienced practitioners blunted the effect seen in randomized trials. In addition, we identified an association of clipping with a higher rate of discharge to rehabilitation. Although disposition does not necessarily reflect functional outcome, some investigators have used it as such.[2, 3] The definitive comparison of the two techniques on functional outcomes, however, can only be done in prospective registries. In this direction, the NeuroPoint Alliance has created the first module for a cerebrovascular registry, with results expected in the near future.[18]

Our study has several limitations common to administrative databases. Residual confounding could account for some of the observed associations. However, this is minimized to the extent that we are using a good instrument for coiling. The F statistic in our analysis suggests a strong instrument. In addition, coding inaccuracies will undoubtedly occur and can affect our estimates. However, several reports have demonstrated that coding for aneurysm and cerebrovascular disease has shown nearly perfect association with medical record review [19, 20]. Although SPARCS includes all hospitals from the entire New York State, the generalization of this analysis to the entire US population is uncertain. SPARCS does not provide any clinical information on the structure, size, or location of the aneurysms, which are important factors in cerebrovascular neurosurgery. However, the use of the instrumental variable analysis is attempting to control for unknown confounders such as these.

Additionally, we were lacking post-hospitalization, and long-term data on our patients. Quality metrics (i.e. modified Rankin score) are also not available through this database, and therefore we cannot compare the two treatment techniques on these outcomes. Although discharge to home does not always indicate a good outcome, discharge status has been shown [21] to correlate well with modified Rankin Scale score and provides important insight into differences within and between treatment modalities. Finally, causality cannot be definitively established based on observational data, despite the use of advanced techniques, such as the instrumental variable analysis.

## Conclusions

Despite the widespread use of coiling in the treatment of ruptured cerebral aneurysms, there is still considerable debate about the relative effectiveness of surgical clipping and endovascular coiling in real world practice. Using a comprehensive all-payer cohort of patients in New York State with aneurysmal SAH we did not identify an association of treatment method with mortality, LOS, or 30-day readmission. Clipping was associated with higher rate of discharge to rehabilitation. Our results were robust when considering several advanced observational techniques to account for measured and unmeasured confounders.

## Supporting Information

S1 Table Coding definitions. (DOC)

## References

[1] Bekelis K, Goodney RP, Dzebisashvili N, Goodman DC, Bronner KK. Variation in the Care of Surgical Conditions: Cerebral Aneurysms. Lebanon, NH: 2014. 36454935

[2] Zacharia BE, Bruce SS, Carpenter AM, Hickman ZL, Vaughan KA, Richards C, et al Variability in outcome after elective cerebral aneurysm repair in high-volume academic medical centers. Stroke. 2014;45(5):1447–52. 10.1161/STROKEAHA.113.004412 24668204

[3] Zacharia BE, Ducruet AF, Hickman ZL, Grobelny BT, Badjatia N, Mayer SA, et al Technological advances in the management of unruptured intracranial aneurysms fail to improve outcome in New York state. Stroke. 2011;42(10):2844–9. 10.1161/STROKEAHA.111.619767 21852601

[4] Molyneux A, Kerr R, Stratton I, Sandercock P, Clarke M, Shrimpton J, et al International Subarachnoid Aneurysm Trial (ISAT) of neurosurgical clipping versus endovascular coiling in 2143 patients with ruptured intracranial aneurysms: a randomised trial. Lancet. 2002;360(9342):1267–74. 12414200 10.1016/s0140-6736(02)11314-6

[5] Harbaugh RE, Heros RC, Hadley MN. More on ISAT. Lancet. 2003;361(9359):783–4. 10.1016/S0140-6736(03)12641-4 12620761

[6] McDougall CG, Spetzler RF, Zabramski JM, Partovi S, Hills NK, Nakaji P, et al The Barrow Ruptured Aneurysm Trial. J Neurosurg. 2012;116(1):135–44. 10.3171/2011.8.JNS101767 22054213

[7] Qureshi AI, Vazquez G, Tariq N, Suri MF, Lakshminarayan K, Lanzino G. Impact of International Subarachnoid Aneurysm Trial results on treatment of ruptured intracranial aneurysms in the United States. Clinical article. J Neurosurg. 2011;114(3):834–41. 10.3171/2010.6.JNS091486 20653392

[8] Health NYSDo. Statewide Planning and Research Cooperative System (SPARCS) 2015 [cited 2015 February 13]. Available from: https://www.health.ny.gov/statistics/sparcs/.

[9] Staiger D, Stock JH. Instrumental Variables Regression with Weak Instruments. Econometrica. 1997;65(3):557–86.

[10] Neuman MD, Rosenbaum PR, Ludwig JM, Zubizarreta JR, Silber JH. Anesthesia technique, mortality, and length of stay after hip fracture surgery. JAMA. 2014;311(24):2508–17. 10.1001/jama.2014.6499 25058085 PMC4344128

[11] Tan HJ, Norton EC, Ye Z, Hafez KS, Gore JL, Miller DC. Long-term survival following partial vs radical nephrectomy among older patients with early-stage kidney cancer. JAMA. 2012;307(15):1629–35. 10.1001/jama.2012.475 22511691 PMC3864575

[12] Xian Y, Holloway RG, Chan PS, Noyes K, Shah MN, Ting HH, et al Association between stroke center hospitalization for acute ischemic stroke and mortality. JAMA. 2011;305(4):373–80. 10.1001/jama.2011.22 21266684 PMC3290863

[13] Garabedian LF, Chu P, Toh S, Zaslavsky AM, Soumerai SB. Potential bias of instrumental variable analyses for observational comparative effectiveness research. Ann Intern Med. 2014;161(2):131–8. 10.7326/M13-1887 25023252

[14] Foster EM. Instrumental Variables for logistic regression: an illustration. Soc Sci Res. 1997;26:287–504.

[15] Molyneux AJ, Kerr RS, Birks J, Ramzi N, Yarnold J, Sneade M, et al Risk of recurrent subarachnoid haemorrhage, death, or dependence and standardised mortality ratios after clipping or coiling of an intracranial aneurysm in the International Subarachnoid Aneurysm Trial (ISAT): long-term follow-up. Lancet Neurol. 2009;8(5):427–33. 10.1016/S1474-4422(09)70080-8 19329361 PMC2669592

[16] Jalbert JJ, Nguyen LL, Gerhard-Herman MD, Jaff MR, White CJ, Rothman AT, et al Outcomes After Carotid Artery Stenting in Medicare Beneficiaries, 2005 to 2009. JAMA Neurol. 2015;1 12 [Epub ahead of print]. 10.1001/jamaneurol.2014.3638 25580726

[17] Feasby TE, Kennedy J, Quan H, Girard L, Ghali WA. Real-world replication of randomized controlled trial results for carotid endarterectomy. Arch Neurol. 2007;64(10):1496–500. 17923633 10.1001/archneur.64.10.1496

[18] NeuroPoint Alliance. The National Neurosurgery Quality and Outcomes Database (N²QOD) 2015 [cited 2015 January 15]. Available from: http://www.neuropoint.org/NPA N2QOD.html.

[19] Kokotailo RA, Hill MD. Coding of stroke and stroke risk factors using international classification of diseases, revisions 9 and 10. Stroke. 2005;36(8):1776–17781. 16020772 10.1161/01.STR.0000174293.17959.a1

[20] Tirschwell DL, Longstreth WTJ. Validating administrative data in stroke research. Stroke. 2002;33(10):2465–70. 12364739 10.1161/01.str.0000032240.28636.bd

[21] Bonita R, Beaglehole R. Recovery of motor function after stroke. Stroke. 1988;19(12):1497–500. 3201508 10.1161/01.str.19.12.1497

