# Supplementary material for: Surgical Clipping versus Endovascular Intervention for the Treatment of Subarachnoid Hemorrhage Patients in New York State
Source: PLoS One. 2015 Sep 11;10(9):e0137946. doi: 10.1371/journal.pone.0137946 (PMC4567333; doi:10.1371/journal.pone.0137946)
Supplement: S1 Table — (DOC) [file pone.0137946.s001.doc]

**Supplemental Material**

**Surgical clipping versus endovascular intervention for the treatment of subarachnoid hemorrhage patients in New York State**

Kimon Bekelis, M.D. ^1^, Symeon Missios, M.D. ^2^, Shannon Coy, B.S. ^3^, Redi Rahmani, B.S. ^3^, Robert J. Singer, M.D. ^1^, Todd A. MacKenzie, Ph.D. ^4,5,6^

^1^Section of Neurosurgery, Dartmouth-Hitchcock Medical Center, Lebanon, NH

^2^ Department of Neurosurgery, Louisiana State University Health Sciences Center, Shreveport, LA

^3^Geisel School of Medicine at Dartmouth, Hanover, NH

^4^Department of Medicine, Dartmouth-Hitchcock Medical Center, Lebanon, NH

^5^Department of Community and Family Medicine, Dartmouth-Hitchcock Medical Center, Lebanon, NH

^6^The Dartmouth Institute for Health Policy and Clinical Practice, Lebanon, NH

**Table of Contents**

SUPPLEMENTARY TABLES…………………………………………………………………………………………………………………………...3

Table S1: Coding definitions………………..…………...………………………………………………………………………………………..………3

| **Table S1. Coding definitions** | | | |
| --- | --- | --- | --- |
| **GROUP** | **CATEGORY** | **CODES** | **NOTES/CODING RULES** |
| **Diagnoses** | **Ruptured aneurysm** | **437.3** | *ICD-9 Diagnosis codes* |
|  |  |  |  |
| **Procedures** | **Clipping** | **39.51** | *ICD-9 Procedure codes* |
|  | **Coiling** | **39.52 (should also have a code 88.41 and no 39.51 during the same hospitalization), 39.72, 39.75, 39.76** | *ICD-9 Procedure codes* |
| **Independent Variables** |  | | |
|  |  |  |  |
|  | Transient ischemic attack | **435**  **435.8**  **435.9** | *ICD-9 Diagnosis codes* |
|  | Ischemic Stroke | **434.91**  **434.11**  **434.01** | *ICD-9 Diagnosis codes* |
|  | Coronary artery disease | **410.xx**  **411.xx**  **412**  **413**  **413.x**  **414**  **414.xx** |  |
|  | Chronic pulmonary disease | **490**  **491.xx**  **492.x**  **493.xx**  **494.x**  **495**  **496** | *ICD-9 Diagnosis codes* |
|  | Congestive heart failure | **402.xx**  **404.xx**  **428.xx**  **425.xx** |  |
|  | Diabetes | **250.xx** |  |
|  | Coagulopathy | **286.x**  **287.1**  **287.3**  **287.4**  **287.5** |  |
|  | Chronic renal failure | **403.11**  **403.91**  **404.12**  **404.92**  **585**  **586**  **V42.0**  **V45.1**  **V56.0**  **V56.8** |  |
|  | Hypertension | **401.x**  **402.xx**  **403.xx**  **404.xx**  **405.xx** |  |
|  | Hypercholesterolemia | **272.0**  **272.1**  **272.2 272.3**  **272.4** |  |
|  | Smoking | **305.1**  **989.84**  **V15.82** | *ICD-9 Diagnosis codes* |
|  | Obesity | **278.00**  **278.01** | *ICD-9 Diagnosis codes* |
|  | Alcohol abuse | **291.xx**  **303.9x**  **305.0x**  **V113** |  |
|  | Peripheral vascular disease | **440.xx**  **441.2**  **441.4**  **441.7**  **441.9**  **443.xx**  **447.1**  **557.1**  **557.9** |  |
